# Isomer-Specific Effects of *cis*-9,*trans*-11- and *trans*-10,*cis*-12-CLA on Immune Regulation in Ruminal Epithelial Cells

**DOI:** 10.3390/ani11041169

**Published:** 2021-04-19

**Authors:** Chunlei Yang, Binna Zhu, Shijie Ye, Zhengwei Fu, Jinjun Li

**Affiliations:** 1 College of Biotechnology and Bioengineering, Zhejiang University of Technology, Hangzhou 310014, China; chunleiyang@zjut.edu.cn (C.Y.); bnzhu97@163.com (B.Z.); yeshijie0423@163.com (S.Y.); 2 Institute of Food Sciences, Zhejiang Academy of Agricultural Sciences, Hangzhou 310021, China

**Keywords:** *cis*-9,*trans*-11-CLA, *trans*-10,*cis*-12-CLA, immunoregulation, lipid metabolism, ruminal epithelial cells

## Abstract

**Simple Summary:**

The significant contribution of rumen microbiota to the balance of the innate immunity of rumen epithelium has been extensively verified. As the natural rumen microbial metabolites, information regarding the immunoprotective effects of different conjugated linoleic acid (CLA) isomers on ruminal epithelial cells (RECs) is limited. In this study, the 100 μM *trans*-10,*cis*-12-CLA exerted better anti-inflammatory effects than the *cis*-9,*trans*-11-CLA by significantly downregulating the expression of genes related to inflammation, cell proliferation and migration in RECs upon lipopolysaccharide (LPS) stimulation. The *trans*-10,*cis*-12-CLA, but not *cis*-9,*trans*-11-CLA, significantly suppressed the biological signals of gene ontology (GO) terms’ response to lipopolysaccharide, the regulation of signal transduction and cytokine production and the Kyoto Encyclopedia of Genes and Genomes (KEGG) pathways NF-κB, chemokine, NOD-like receptor, Hippo, PI3K-Akt, TGF-β and Rap1 signaling in RECs upon LPS stimulation. Furthermore, pretreatment with *trans*-10,*cis*-12-CLA significantly reduced the expression of lipogenic genes and the biosynthesis of the unsaturated fatty acid pathway in RECs compared with the LPS group, however, *cis*-9,*trans*-11-CLA exhibited the opposite results. These results suggest the distinct isomer differences of CLA in the regulation of inflammatory responses and adipocytokine signaling in RECs and will provide important references for determining their target use in the future.

**Abstract:**

In this study, we used transcriptomics and qPCR to investigate the potential immunoprotective effects of different conjugated linoleic acid (CLA) isomers, the natural rumen microbial metabolites, on lipopolysaccharide (LPS)-induced inflammation of ruminal epithelial cells (RECs) in vitro. The results showed that 100 μM *trans*-10,*cis*-12-CLA exerted higher anti-inflammatory effects than *cis*-9,*trans*-11-CLA by significantly downregulating the expression of genes related to inflammation, cell proliferation and migration in RECs upon LPS stimulation. Transcriptomic analyses further indicated that pretreatment with *trans*-10,*cis*-12-CLA, but not *cis*-9,*trans*-11-CLA, significantly suppressed the biological signals of GO terms’ response to LPS, the regulation of signal transduction and cytokine production and KEGG pathways NF-κB, chemokine, NOD-like receptor, Hippo, PI3K-Akt, TGF-β and Rap1 signaling in RECs upon LPS stimulation. Furthermore, pretreatment with *trans*-10,*cis*-12-CLA significantly reduced the expression of lipogenic genes and the biosynthesis of the unsaturated fatty acid pathway in RECs compared with the LPS group, however, *cis*-9,*trans*-11-CLA exhibited the opposite results. These results suggest the distinct isomer differences of CLA in the regulation of inflammatory responses and adipocytokine signaling in RECs and will provide important references for determining their target use in the future.

## 1. Introduction

The ruminal epithelium not only plays a significant role in nutrient absorption and metabolism but also serves as an important barrier to protect hosts against stimuli, such as pathogens and toxins [1]. The accumulated lipopolysaccharide (LPS) derived from the lysis of Gram-negative bacteria within the rumen, especially in ruminal disorders, always presents great challenges for the ruminal epithelium [2]. The interaction of the ruminal epithelium and microbiota plays a key role in sustaining the balance of the inflammatory response and immune tolerance in the rumen [3]. Microbial metabolites have been reported to exert important roles in the integrity of ruminal epithelium, expression of tight junctions, renewal of epithelial cells and immune-response-related signals [4,5].

Conjugated linoleic acid (CLA) is a type of natural ruminal microbial metabolite that forms during the biohydrogenation process within the rumen [6]. It is a mixture of positional and geometrical isomers of linoleic acid with conjugated double bonds, and *cis*-9,*trans*-11-CLA and *trans*-10,*cis*-12-CLA are the most studied bioactive isomers [7]. Various beneficial effects have been found in CLA, such as anticarcinogenic, antiobesogenic, anti-inflammatory and immune-enhancement effects [8,9]. However, which CLA isomer is responsible for the observed benefits is sometimes controversial, with species-, tissue- and cell-type-dependent specific effects reported [10,11,12,13]. In the study of Su et al. [14], the isomers *cis*-9,*trans*-11-CLA impaired intestinal epithelial barrier function in both mice and IPEC-J2 cells, whereas *trans*-10,*cis*-12-CLA did not. However, in the study of Jaudszus et al. [15], *cis*-9,*trans*-11-CLA significantly reduced the release of inflammatory cytokines in stimulated human bronchial epithelial cells and exerted better anti-inflammatory effects than *trans*-10,*cis*-12-CLA. Furthermore, after comparing the effects of CLA isomers on adipose tissue, muscle and liver of mice, Della Casa et al. [13] found that *tran*-10,*cis*-12-CLA effectively reduced fat masses in adipose tissue and increased fatty acid oxidation in muscle but also induced liver steatosis, conversely, *cis*-9,*trans*-11-CLA had no relevant effects on adipose tissue and liver, but it enhanced muscular cell differentiation. Dietary supplementation of ruminants with CLA is a well-known nutrition strategy, which allows the improvement of milk quality and whole-body energy utilization, and the mitigation of the proinflammatory response associated with oxidative stress around the calving period in dairy cows. However, most findings result from a mixture of CLA isomers due to commercial availability [16,17,18], therefore, the information regarding isomer-specific effects on the immunity of rumen are limited. Factors including dietary starch or polyunsaturated fatty acid (PUFA) content, ruminal pH and bacterial activity are suggested to be important regulators driving the abundance of different CLA isomers in rumen, and more *trans*-10,*cis*-12-CLA can be produced when high-grain diets are supplied [6,19]. Therefore, a better understanding of how these specific CLA isomers play a role in regulating inflammatory responses in ruminal epithelium can provide more information for their target enrichment or use in the management of ruminant health.

The function of CLA as a signal to regulate various physiological processes is mostly involved in modulating the expression and activity of peroxisome proliferator-activated receptor gamma (*PPARγ*), given its high similarity with the *PPARγ* ligand [20]. PPARs are members of the nuclear receptor subfamily that exerts pleiotropic effects by affecting the expression of gene networks involved in inflammation, metabolism of lipids and carbohydrates, homeostasis and cell-cycle regulation [21]. Furthermore, CLA isomers have been found to possess the ability to affect lipid metabolism, cell survival and apoptosis, which are considered to be closely associated with inflammatory signaling cascades [20,22,23,24,25]. However, many of the mechanisms are still not fully clarified, and isomer-specific effects are also present [26,27]. RNA plays an important role in various biological processes, and RNA sequencing has emerged as an in-depth study model to explore gene changes and alternative splicing in various cell populations and to detect novel transcripts [28]. Thus, to better understand the potential protective effects of different CLA isomers on ruminal epithelial cells and to elucidate the related regulatory mechanisms, we used the transcriptome sequencing method to investigate the changed gene expression and functions in RECs under inflammatory conditions with either *cis*-9,*trans*-11-CLA or *trans*-10,*cis*-12-CLA pretreatment. We hypothesized that different CLA isomers may protect RECs against inflammation differently.

## 2. Materials and Methods

### 2.1. Cell Treatments

The immortalized ruminal epithelial cell line RECs was cultured in DMEM (Gibco, New York, USA) containing 2% FBS, 1% penicillin/streptomycin and 1% epithelial cell additive at 37 °C with 5% CO_2_, as described previously [29]. When REC monolayers reached 70–80% confluency, the cells were treated without (control group, CON) or with 0.1 μg/mL LPS (from *Escherichia coli* O111:B4, Sigma-Aldrich, Shanghai, China) for 3 h [29] after pretreatment with 50 or 100 μM *cis*-9,*trans*-11- (*c*-9,*t*-11-CLA+LPS group, *c*-9,*t*-11-CLA+LPS) or *trans*-10,*cis*-12-CLA (*t*-10,*c*-12-CLA+LPS group, *t*-10,*c*-12-CLA+LPS) (purity ≥ 96.0%, Sigma-Aldrich, Shanghai, China) for 24 h or not (LPS group, LPS). Six treatments were contained and each treatment had 4 replicates.

### 2.2. RNA Isolation and Real-Time PCR (qPCR) Analysis

Total RNA was extracted from all the treated RECs with RNA PURE KIT (Aidlab Biotechnologies Co., Ltd., Beijing, China) according to the manufacturer’s instructions. cDNA was then synthesized using the PrimeScript RT Reagent Kit (Takara, Dalian, China). qPCR was subsequently performed in an ABI 7500 (Life Technologies, Singapore) with SYBR green using procedures described previously [29]. The results were normalized to glyceraldehyde-3-phosphate dehydrogenase (GAPDH) and tyrosine 3-monooxygenase/tryptophan 5-monooxygenase activation protein zeta (YWHAZ) expression using the 2^^−∆∆Ct^ method. The primers were designed using the Basic Local Alignment Search Tool [BLAST; National Center for Biotechnology Information (NCBI), Bethesda, MD, USA] and are presented in Table S1.

### 2.3. Transcriptome Sequencing

After RNA extraction, RNA purity and concentration were measured by a NanoPhotometer spectrophotometer (IMPLEN, Westlake Village, CA, USA) and Qubit RNA assay kit with a Qubit 2.0 fluorometer (Life Technologies, Carlsbad, CA, USA), respectively. RNA integrity was determined by the RNA Nano 6000 assay kit with the Bioanalyzer 2100 system (Agilent Technologies, Santa Clara, CA, USA). A total of 3 μg RNA per sample was utilized for library preparation using the NEBNext Ultra RNA library prep kit for Illumina (NEB, Ipswich, MA, USA) according to the manufacturer’s instructions. Paired-end sequencing (150 bp) was performed via the Illumina HiSeq 2000 instrument, and a minimum depth of 40 million reads per sample was obtained. The RNA sequencing work was supported by the Beijing Novogene Biological Information Technology Co., Ltd. 

### 2.4. Data Processing and Analysis

The raw data were processed with quality control and mapped to the reference genome and reference gene annotations of *Ovis aries* (ftp://ftp.ensembl.org/pub/release-95/fasta/ovis_aries/ and ftp://ftp.ensembl.org/pub/release-95/gtf/ovis_aries/) using Hisat2v.2.0.5 (accessed on 12 September 2020). The expected number of fragments per kilobase of transcript sequence per million base pairs sequenced (FPKM) of each gene was calculated based on the length of the gene and read count mapped to this gene and was used for the evaluation of the gene expression level. The differential gene expression among the CON, LPS, *c*-9,*t*-11-CLA+LPS and *t*-10,*c*-12-CLA+LPS groups was analyzed using the DESeq2 R package (1.16.1), and the resulting *p*-values were adjusted (*p*_adj_) using Benjamini and Hochberg’s approach to control the false discovery rate. Genes with *p*_adj_ < 0.05 and fold change (FC) > 1.5 were considered differentially expressed genes (DEGs) [30].

### 2.5. Functional Analysis of the DEGs

To better annotate the biological functions of the DEGs, gene ontology (GO) enrichment and Kyoto Encyclopedia of Genes and Genomes (KEGG) pathway analysis of the DEGs was performed using the GOseq R package and KOBAS software, respectively. The GO and KEGG terms with a *p*-value < 0.05 were considered significantly enriched for the DEGs. The protein–protein interaction (PPI) networks for the DEGs were analyzed using the STRING v.10.5 database (http://string-db.org/) (accessed on 12 September 2020) to better understand the relationships between the proteins and genes identified. The hub genes (degree > 5) in the networks were analyzed by Cytoscape. 

### 2.6. Statistical Analysis

The data for qPCR analysis were presented as the means ± SEM. The unpaired Student’s *t*-test was used for comparisons between two groups. *p*-values < 0.05 were considered statistically significant.

## 3. Results

### 3.1. Optimization of the Concentrations of cis-9,trans-11- and trans-10,cis-12-CLA 

**Figure 1:**
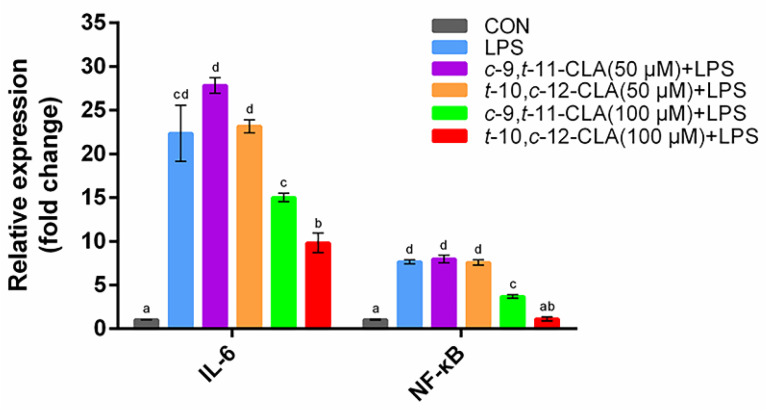
Gene expression of proinflammatory cytokines in ruminal epithelial cells (RECs) upon lipopolysaccharide (LPS) stimulation with pretreatment with 50 or 100 μM of different conjugated linoleic acid (CLA) isomers. The cells were treated without (CON) or with 0.1 μg/mL LPS for 3 h after pretreatment with 50 or 100 μM *cis*-9,*trans*-11-CLA (*c*-9,*t*-11-CLA+LPS) or *trans*-10,*cis*-12-CLA (*t*-10,*c*-12-CLA+LPS) for 24 h or not (LPS). ^a–d^ Bars that share different superscript letters are significantly different from each other (*p* < 0.05).

The qPCR results for measuring the production of the proinflammatory factors *IL-6* and *NF-κB* in RECs after exposure to LPS for 3 h of pretreatment with *cis*-9,*trans*-11- or *trans*-10,*cis*-12-CLA for 24 h at concentrations of 50 and 100 μM, respectively, indicated that the regulation of CLA isomers on the gene expression of *IL-6* and *NF-κB* in RECs was concentration-dependent. Pretreatment with 50 μM *cis*-9,*trans*-11- or *trans*-10,*cis*-12-CLA did not significantly affect *IL-6* and *NF-κB* gene expression in RECs upon LPS stimulation. However, pretreatment with 100 μM *trans*-10,*cis*-12-CLA significantly reduced the gene expression of *IL-6* and *NF-κB* in RECs upon LPS stimulation (*p* < 0.05), while pretreatment with 100 μM *cis*-9,*trans*-11-CLA significantly reduced the gene expression of *NF-κB* in RECs upon LPS stimulation (*p* < 0.05) (Figure 1). Therefore, pretreatment with 100 μM *cis*-9,*trans*-11 or *trans*-10,*cis*-12-CLA for 24 h exhibited better protective results against inflammation in RECs and was selected for further transcriptomics analysis.

### 3.2. Overview of the RNA Sequencing Among Different Treatments 

**Figure 2:**
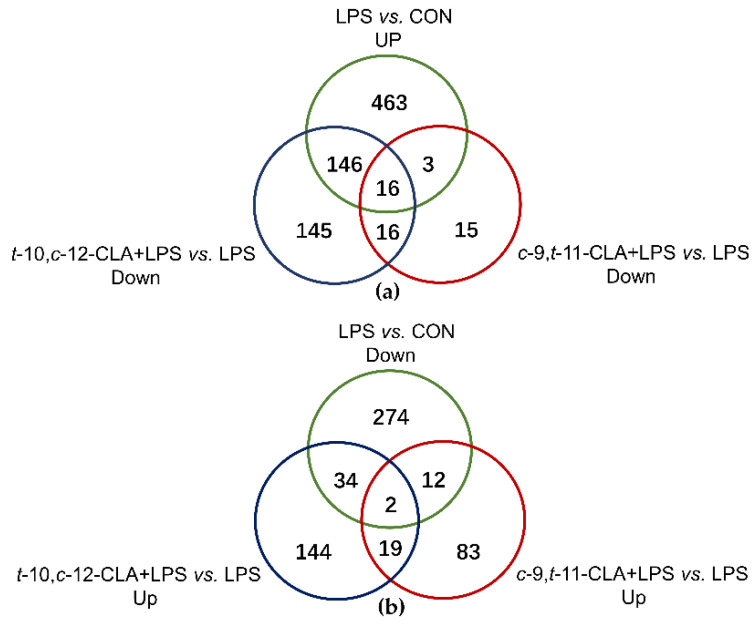
Venn diagrams of the known differentially expressed genes (DEGs) according to the comparisons of different treatments. (**a**) The DEGs that were downregulated by the pretreatment of either *cis*-9,*trans*-11-conjugated linoleic acid (CLA) or *trans*-10,*cis*-12-CLA and upregulated during LPS stimulation. (**b**) The DEGs that were upregulated by the pretreatment of either *cis*-9,*trans*-11-CLA or *trans*-10,*cis*-12-CLA and downregulated during lipopolysaccharide (LPS) stimulation. The cutoff values of the differential expression criteria were fold change (FC) > 1.5 and *p*_adj_ < 0.05.

An average of 50.33 ± 7.93 million raw reads were obtained per sample among all the treatments. After performing data quality, 50.07 ± 7.86 million clean reads were obtained with the contamination, and low-quality reads were removed. Greater than 85.20% of the clean sequencing reads per sample were successfully mapped to the reference genome of *Ovis aries*. After the calculation of FPKM of each sample, a similar gene expression distribution among all the treatments was obtained. After novel genes were removed, 628 genes were significantly upregulated, and 322 genes were downregulated with LPS treatment compared with the expression levels in the CON group (FC > 1.5, *p*_adj_ < 0.05) (Figure 2a,b). One hundred and sixteen genes were significantly upregulated, and 50 genes were downregulated in the *c*-9,*t*-11-CLA+LPS group compared with the LPS group (FC > 1.5, *p*_adj_ < 0.05) (Figure 2a,b). For the treatment of *t*-10,*c*-12-CLA+LPS, 199 genes were significantly upregulated, and 323 genes were downregulated compared with the LPS group (FC > 1.5, *p*_adj_ < 0.05) (Figure 2a,b). Specifically, 19 overlapping genes were significantly upregulated with LPS treatment and downregulated with *c*-9,*t*-11-CLA+LPS treatment, and 162 overlapping genes were significantly upregulated with LPS treatment and downregulated with *t*-10,*c*-12-CLA+LPS treatment (FC > 1.5, *p*_adj_ < 0.05) (Figure 2a). In addition, 16 of the overlapping genes were shared between *c*-9,*t*-11-CLA+LPS vs. LPS downregulation and *t*-10,*c*-12-CLA+LPS vs. LPS downregulation; these genes were upregulated with LPS stimulation (FC > 1.5, *p*_adj_ < 0.05) (Figure 2a). Fourteen and 36 overlapping genes were significantly upregulated by treatment with *c*-9,*t*-11-CLA+LPS and *t*-10,*c*-12-CLA+LPS compared with the LPS group and downregulated by LPS treatment compared with the CON group, respectively (FC > 1.5, *p*_adj_ < 0.05) (Figure 2b).

### 3.3. trans-10,cis-12-CLA Exhibited a Better Effect on Preventing Inflammatory Responses in RECs upon LPS Stimulation than cis-9,trans-11-CLA

**Figure 3:**
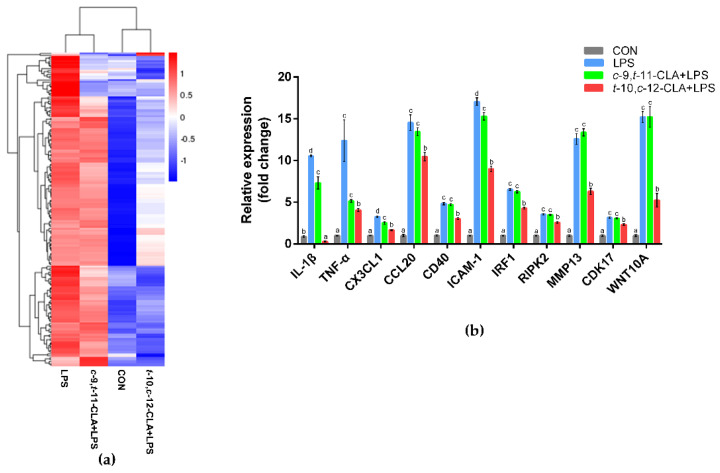
Effect of pretreatment with different conjugated linoleic acid (CLA) isomers on preventing inflammatory responses in lipopolysaccharide (LPS) −stimulated ruminal epithelial cells (RECs). (**a**) Hierarchical clustering analysis of the overlapping differentially expressed genes (DEGs) that were downregulated by CLA isomer pretreatment and upregulated during LPS stimulation. The color code represents genes expressed at low (blue) and high (red) levels. (**b**) Gene expression levels of inflammation−related factors among the different groups. ^a−d^ Bars that share different superscript letters are significantly different from each other (*p* < 0.05).

According to the hierarchical cluster analysis of the overlapping DEGs that were downregulated in the *c*-9,*t*-11-CLA+LPS or *t*-10,*c*-12-CLA+LPS group and upregulated in the LPS group, a clear separation of the CON and *t*-10,*c*-12-CLA+LPS groups from the LPS and *c*-9,*t*-11-CLA+LPS groups was observed (Figure 3a). The qPCR results further confirmed that pretreatment with *t*-10,*c*-12-CLA significantly reduced the expression of the proinflammatory cytokines *IL-1β* and *TNF-α*, chemokines *CX3CL1* and *CCL20*, members of the *TNF* receptor superfamily *CD40*, intercellular cell adhesion molecule *ICAM-1*, interferon regulatory factor *IRF1* and receptor-interacting serine/threonine kinase 2 (*RIPK2*) upon LPS stimulation (*p* < 0.05) and prevented stimulus-induced increases in cell proliferation and migration. Specifically, the gene expression of *CDK17*, *WNT10A* and *MMP13* was significantly reduced upon LPS stimulation (*p* < 0.05) (Figure 3b). However, pretreatment with *c*-9,*t*-11-CLA only significantly suppressed the expression of *IL-1β*, *CX3CL1* and *ICAM-1* upon LPS stimulation (Figure 3b).

**Figure 4:**
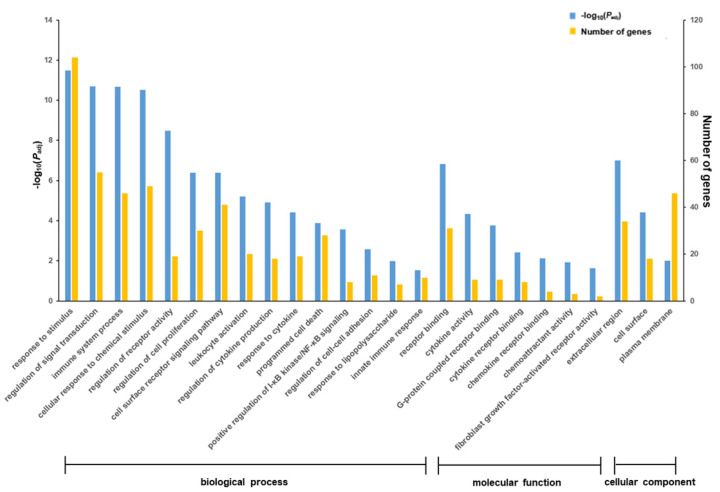
Effect of *trans*-10,*cis*-12-conjugated linoleic acid (CLA) pretreatment on the gene ontology (GO) terms in ruminal epithelial cells (RECs) upon lipopolysaccharide (LPS) stimulation. The differentially expressed genes (DEGs) used for functional annotation were significantly downregulated in the *t*-10,*c*-12-CLA+LPS group and upregulated in the LPS group.

**Figure 5:**
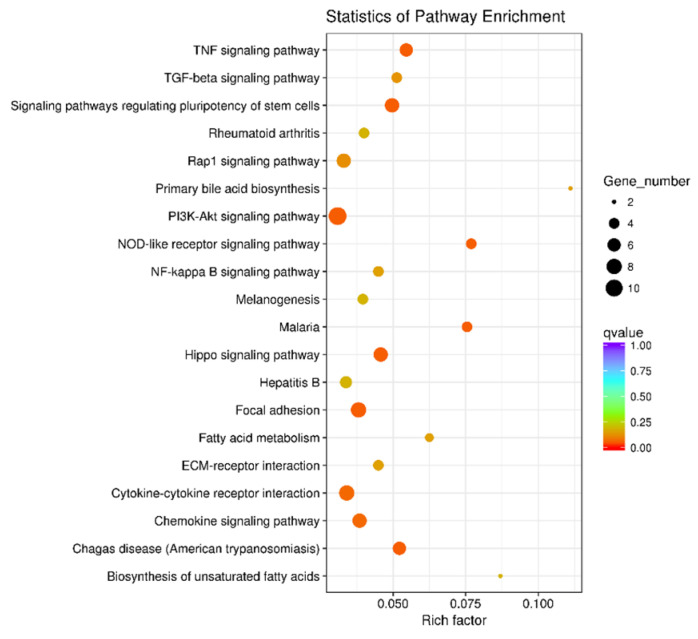
Effect of *trans*-10,*cis*-12-conjugated linoleic acid (CLA) pretreatment on the Kyoto Encyclopedia of Genes and Genomes (KEGG) pathways in ruminal epithelial cells (RECs) upon lipopolysaccharide (LPS) stimulation. The differentially expressed genes (DEGs) used for functional annotation were significantly downregulated in the *t*-10,*c*-12-CLA+LPS group and upregulated in the LPS group. The circle size indicates the number of enriched genes, and the circle color indicates the *p*_adj_ value.

The GO and KEGG analysis of the overlapping DEGs further confirmed that pretreatment with *t*-10,*c*-12-CLA significantly downregulated the inflammatory-related biological processes of the response to LPS and cytokine, regulation of receptor activity, signal transduction and cytokine production, leukocyte activation and immune system processes, positive regulation of I-κB kinase/NF-κB signaling, molecular functions of cytokine, chemokine and G-protein-coupled receptor binding, chemoattractant activity and cellular components of cell surface and plasma membrane (*p* < 0.05) (Figure 4), as well as the pathways of TNF, NF-κB, chemokine, NOD-like receptor signaling and cytokine–cytokine receptor interaction, which were significantly upregulated upon LPS stimulation (*p* < 0.05) (Figure 5). In addition, the stimulus-induced increase in cell proliferation and migration-related biological processes of the regulation of cell proliferation and cell–cell adhesion, the molecular function of fibroblast growth factor-activated receptor activity (Figure 4), and the pathways of Hippo, PI3K-Akt, TGF-β, Rap1 signaling and focal adhesion (Figure 5) were also significantly downregulated by pretreatment with *t*-10,*c*-12-CLA compared with the LPS group (*p* < 0.05). However, the GO terms and KEGG pathways that were significantly downregulated by pretreatment with *c*-9,*t*-11-CLA compared with LPS stimulation were less related to inflammatory regulation, and only the TNF signaling pathway was significantly enriched (*p* < 0.05) (Figure S1).

### 3.4. cis-9,trans-11-CLA and trans-10,cis-12-CLA Affected TLR4 and PPARγ Signaling in an Isomer-Specific Manner

**Figure 6:**
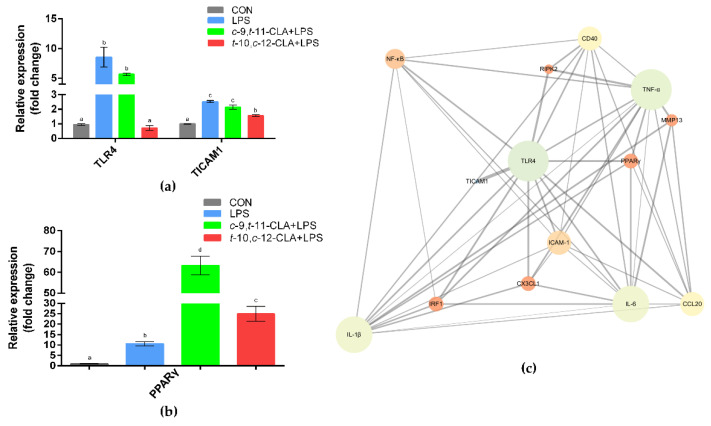
Protective effects of conjugated linoleic acid (CLA) isomers on ruminal epithelial cells (RECs) upon lipopolysaccharide (LPS) stimulation with regard to *TLR4*- and PPARγ-related signaling. The gene expression levels of *TLR4* and *TICAM1* (**a**) and *PPARγ* (**b**) among the different groups. ^a–d^ Bars that share different superscript letters are significantly different from each other (*p* < 0.05). (**c**) Protein–protein interaction (PPI) network of *TLR4* and *PPARγ* with differentially expressed genes (DEGs) that are related to inflammatory responses. The node size and color represent the number of degrees and the clustering coefficient (blue indicates low values, red indicates high values), respectively, and the edge represents the interaction (the thicker edge indicates the lower betweenness).

*TLR4* is the most well-known receptor of LPS on epithelial cells. Stimulation of RECs with LPS significantly increased the gene expression level of *TLR4*, but pretreatment with 100 μM *t*-10,*c*-12-CLA significantly reversed this effect (*p* < 0.05) (Figure 6a). The gene expression of *TICAM1*, which is a Toll-like receptor adaptor molecule, was also significantly reduced by pretreatment with 100 μM *t*-10,*c*-12-CLA upon LPS stimulation (*p* < 0.05) (Figure 6a). However, pretreatment with 100 μM *c*-9,*t*-11-CLA yielded no significant effects on the expression of *TLR4* and *TICAM1* in RECs, which was consistent with its minor anti-inflammatory effects on RECs. The expression of *PPARγ*, which is involved in suppressing inflammatory responses, was significantly increased by pretreatment with either *c*-9,*t*-11-CLA or *t*-10,*c*-12-CLA compared with the LPS group (*p* < 0.05) (Figure 6b). PPI analysis of the DEGs among the *t*-10,*c*-12-CLA+LPS and LPS groups further indicated that *TLR4*, *PPARγ*, *IL-6*, *TNF-α*, *IL-1β*, *CD40*, *ICAM-1*, *NF-κB*, *IRF1*, *CCL20* and *CX3CL1* were identified as hub genes (degree > 5) and showed close interactions (Figure 6c).

### 3.5. cis-9,trans-11-CLA and trans-10,cis-12-CLA Affected Adipocytokine Signaling in an Isomer-Specific Manner

**Figure 7:**
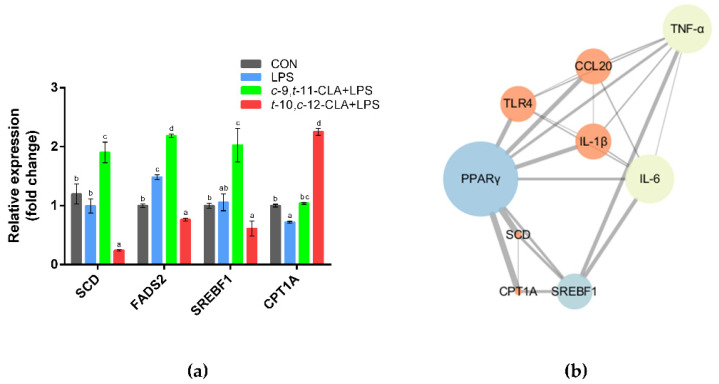
Effects of pretreatment with different conjugated linoleic acid (CLA) isomers on the expression of genes related to lipid metabolism in ruminal epithelial cells (RECs). (**a**) Gene expression levels of *SCD*, *FADS2*, *SREBF1* and *CPT1A* among different groups. ^a–d^ Bars that share different superscript letters are significantly different from each other (*p* < 0.05). (**b**) Protein–protein interaction (PPI) network of the differentially expressed genes (DEGs) involved in lipid metabolism and inflammatory responses. The node size and color represent the number of degrees and the clustering coefficient (blue indicates low values, red indicates high values), respectively, and the edge represents the interaction (the thicker edge indicates the lower betweenness).

**Figure 8:**
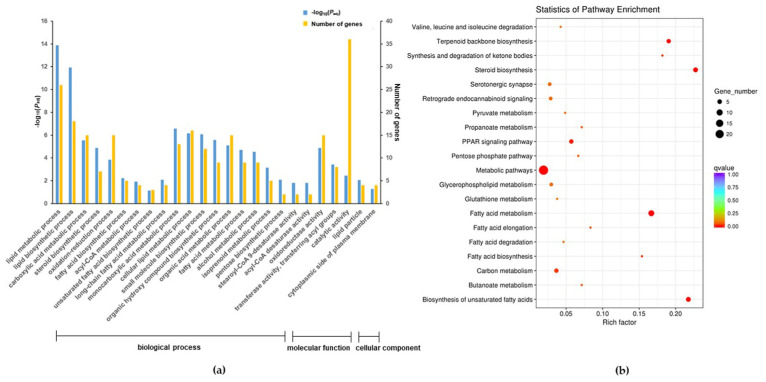
The gene ontology (GO) terms (**a**) and Kyoto Encyclopedia of Genes and Genomes (KEGG) pathways (**b**) for the differentially expressed genes (DEGs) that were significantly upregulated in the *c*-9,*t*-11-conjugated linoleic acid (CLA)+lipopolysaccharide (LPS) group compared with the LPS group. The circle size indicates the number of enriched genes, and the circle color indicates the *p*_adj_ value.

The qPCR results indicated that pretreatment with *c*-9,*t*-11- and *t*-10,*c*-12-CLA both significantly upregulated the expression level of carnitine palmitoyltransferase 1A (*CPT1A*) in RECs upon LPS stimulation (*p* < 0.05) (Figure 7a). Although *t*-10,*c*-12-CLA significantly downregulated the expression of the genes encoding stearoyl-CoA desaturase (*SCD*) and fatty acid desaturase 2 (*FADS2*), *c*-9,*t*-11-CLA significantly upregulated the expression of the genes encoding *SCD*, *FADS2* and sterol regulatory element binding transcription factor 1 (*SREBF1*) compared with the LPS group (*p* < 0.05) (Figure 7a). KEGG and GO analyses further indicated that the DEGs that were downregulated in the *t*-10,*c*-12-CLA+LPS group, compared to the LPS group, were significantly enriched in the pathways of fatty acid metabolism and biosynthesis of unsaturated fatty acids (Figure 5), whereas the DEGs that were upregulated in the *c*-9,*t*-11-CLA+LPS group were significantly enriched in the signals of molecular functions of stearoyl-CoA 9-desaturase and acyl-CoA desaturase activity (Figure 8a), as well as the pathways of fatty acid metabolism and the biosynthesis of unsaturated fatty acids (Figure 8b). PPI analysis of the DEGs regarding adipocytokine and inflammatory cytokine signals further identified *SREBF1*, *PPARγ*, *TLR4*, *IL-6*, *TNF-α*, *IL-1β* and *CCL20* as hub genes (degree > 5), which also showed close interactions (Figure 7b).

## 4. Discussion

Dietary supplementation of CLA for ruminants has been regarded as a regular nutrition strategy due to the various benefits of CLA *in vivo*, but most of them are a mixture of both CLA isomers due to commercial availability [16,17,18]. Information about the isomer-specific effects of CLA on the physiology and immunity of rumen are limited. However, a growing number of studies have indicated that each isomer sometimes exerts different physiological and immunoregulatory effects, with species-, tissue- and cell-type-dependent effects reported [10,11,12]. In the study of Dipasquale et al. [31], the *trans*-10,*cis*-12-CLA was reported to exert a more balanced and efficient protective effect on bovine mammary epithelial cells against LPS stimulation than *cis*-9,*trans*-11-CLA. However, in the study of Jaudszus [15], *cis*-9,*trans*-11-CLA showed better anti-inflammatory effects in stimulated human bronchial epithelial cells than *trans*-10,*cis*-12-CLA. The 2 isomers of CLA even showed contradictory immunomodulatory effects on bovine peripheral blood mononuclear cells and mammary gland epithelial cells [32,33]. We demonstrated that supplementation with 100 μM *trans*-10,*cis*-12-CLA inhibited the production of the proinflammatory cytokines *IL-1β*, *IL-6* and *TNF-α*; chemokines *CX3CL1* and *CCL20*; members of the *TNF* receptor superfamily *CD40*; interferon regulatory factor *IRF1*; and intercellular cell adhesion molecule *ICAM-1* in RECs, in contrast, *cis*-9,*trans*-11-CLA only suppressed the production of *IL-1β*, *CX3CL1* and *ICAM-1* upon LPS stimulation. As the major component of the outer membrane of Gram-negative bacteria, LPS can induce many intracellular inflammatory responses, including the expression and release of proinflammatory cytokines, chemokines, interferon and cell adhesion molecules [34]. Therefore, the 100 μM *trans*-10,*cis*-12-CLA exhibited better protective effects on RECs against LPS-induced inflammation compared with the *cis*-9,*trans*-11-CLA in the present study, but in vivo studies should be performed to support these findings in the future.

LPS stimulation results in the initiation of many intracellular inflammation-related signaling pathways [35]. In the present study, our results found that *trans*-10,*cis*-12-CLA pretreatment suppressed the TNF, NF-κB and chemokine signaling pathways and the cytokine–cytokine receptor interaction in RECs induced by LPS stimulation. Evidence indicates that the physiological connections between proinflammatory cytokines and NF-κB largely contribute to inflammation and the immune response and can regulate each other to a certain extent. For example, *TNF-α*, *IL-1β* and *IL-6* are inducers of cytoplasmic NF-κB activation and lead to its subsequent translocation into the nucleus, where it further stimulates the expression and release of proinflammatory cytokines, thereby aggravating inflammatory responses [36,37]. The ability of NF-κB to regulate the transcription of *ICAM-1*, thereby enhancing vascular adhesion and the activation of inflammatory cells, as well as inducing chemokine-signaling that can potently attract and recruit more T cells and monocytes to the inflammatory site, has also been proven [38,39]. Therefore, CLA may protect RECs against LPS-induced inflammation by inhibiting the NF-κB-mediated transcriptional induction of proinflammatory cytokines, adhesion molecules and chemokine signaling.

In addition, we observed that pretreatment with *trans*-10,*cis*-12-CLA suppressed the LPS-induced increase in REC proliferation and migration, whereas *cis*-9,*trans*-11-CLA did not exhibit this effect. The isomer-specific effect was also found in the study of Lee et al. [40], in which *trans*-10,*cis*-12-CLA suppressed cell proliferation and induced apoptosis in human colorectal cancer, whereas *cis*-9,*trans*-11-CLA showed no such effect. The qPCR and transcriptome results further indicated that the inhibitory effects were based on the mechanisms of downregulating the mRNA expression of *CDK17*, *WNT10A* and *MMP13,* as well as the molecular function of fibroblast growth factor-activated receptor activity; pathways of Hippo, PI3K-Akt, TGF-β, Rap1 signaling and focal adhesion in RECs, which are positively involved in cell proliferation and migration [41,42,43,44,45,46,47,48,49]. Accumulating evidence suggests that inflammatory mediators, especially cytokines, such as *TNF-α*, *IL-1β* and *IL-6*, play important roles in regulating the initiation and progression of cell proliferation, survival, migration and apoptosis via their effects on epithelial signaling pathways, such as signal transducer and activator of transcription 3 (STAT3), c-Jun NH2-terminal kinase (JNK), mitogen-activated protein kinase (MAPK) and NF-κB [25,50]. For example, the activation of NF-κB can promote epithelial cell survival and proliferation by stimulating the transcription of proliferation-regulating genes, such as *cyclin D1* and *c-Myc* [51], as well as genes involved in cell migration and invasion, such as *MMP2* and *MMP9* [52]. Therefore, *trans*-10,*cis*-12-CLA might mediate transcriptome reprogramming of RECs to prevent stimulus-induced cell proliferation and migration.

In innate immunity, the specific host pathogen recognition receptors (PRRs) that consist of an array of sensors in the plasma, plasma membranes and host cytosol are often responsible for recognizing conserved pathogen-associated molecular patterns, including LPS [53]. Toll receptors (TLRs) and NOD-like receptors (NLRs) are two types of PRRs that localize at the cell surface or within endosomes and the intracellular cytosol, respectively [54]. TLR4 is the major recognition receptor of LPS, and the significant role of TLR4-mediated intracellular signaling as an inducer of various transcription factors, including NF-κB, AP1, STAT1 and IRFs, which are involved in the initiation and regulation of inflammatory responses, has been extensively verified [55]. Emerging evidence has found that NLR-mediated signaling is another significant factor involved in inflammatory responses upon LPS stimulation [56]. For example, the NLR Nod2 plays an essential role in the activation of NF-κB and MAPK signaling pathways and robust production of proinflammatory cytokines and chemokines in neutrophils in response to bacterial infection [57]. LPS stimulation can also induce the activation of the NLR-family pyrin domain-containing 3 (NLRP3) inflammasome, leading to pro-IL-1β expression and *IL-1β* maturation [58]. Simultaneously, the cooperation of Nod1 and Nod2 with TLRs shapes the host response to bacterial stimuli [59]. In the present study, the *TLR4* expression, induction of NLRs and NF-κB signaling pathways and the expression of *RIPK2*, which is recruited by the Nod1 and Nod2 receptors and contributes to NF-κB-mediated inflammatory signaling [60], were significantly upregulated in RECs after LPS stimulation and were significantly downregulated by pretreatment with *trans*-10,*cis*-12-CLA upon LPS stimulation. Simultaneously, *TLR4* exhibited a close correlation with *NF-κB* as well as proinflammatory cytokines, chemokines, interferon and cell adhesion molecules, according to PPI analysis, indicating that the protective effects of CLA on RECs against inflammation induced by LPS stimulation likely occur through the NLR or TLR4-NF-κB-mediated signaling pathway.

The PPARγ-dependent pathway is one of the most well-known immune regulation mechanisms of CLA due to the high similarity of CLA with PPAR ligands [20]. The important role of *PPARγ* in the control of the inflammatory response is largely dependent on its ability to suppress the activities of many transcription factors, such as NF-κB, STAT and AP1 that are involved in the immune process [61]. In the present study, RECs pretreated with either *trans*-10,*cis*-12-CLA or *cis*-9,*trans*-11-CLA expressed more *PPARγ* following stimulation with LPS, which is consistent with previous findings [31,62]. Jaudszus [15] also found that, when treated stimulated bronchial epithelial cells with CLA, IL-8 production was significantly decreased and mediated by the activation of *PPARγ* given that the effect was restored when blocking the activity of *PPARγ* with its selective antagonist was used. Our study revealed that *PPARγ* expression exhibited a close correlation with the expression of *TNF-α*, *IL-1β*, *IL-6*, *CCL20* and *TLR4*. Many studies have suggested that the activation of *PPARγ* can attenuate LPS-induced TLR4 expression and decrease the expression of proinflammatory cytokines and chemokines [63,64,65]. Therefore, CLA also exhibited potent anti-inflammatory effects on RECs though PPARγ-dependent downregulation of inflammatory responses.

In addition to the direct regulatory effects of CLA on inflammatory processes, we also observed the significant role of CLA in lipid metabolism. Accumulating evidence suggests that lipid metabolism and inflammatory responses are coordinately regulated at multiple levels within the body [66]; for example, the promotion of fatty acid oxidation (FAO) positively correlated with the relief of inflammation via AMPK or PPARδ-dependent signaling [24], and endothelial loss of FAO stimulated the dysfunction of endothelial cells with barrier disruption and leukocyte infiltration by increasing endothelial oxidative stress [67]. In the present study, pretreatment with *trans*-10,*cis*-12-CLA or *cis*-9,*trans*-11-CLA significantly promoted the gene expression of *CPT1A*, which is a rate-limiting enzyme of the FAO process [68], which was consistent with a previous study that demonstrated the positive role of CLA in FAO [23]. The interplay among the expression of *CPT1A*, *PPARγ* and inflammatory cytokines according to the PPI analysis in our study indicated the anti-inflammatory effects of CLA, which probably occur through a PPAR-dependent FAO shift mechanism. Zhao et al. [69] also found that the activated PPARγ signaling pathway could drive the FAO process in dendritic cells by upregulating *CPT1A* expression, and the FAO shift suppressed *IL-6* and *IL-12* expression. Simultaneously, our study found that pretreatment with *trans*-10,*cis*-12-CLA reduced the expression of genes involved in lipogenesis, including *SCD* and *FADS2*, whereas *cis*-9,*trans*-11-CLA exerted the opposite effects. Although CLA is known to be involved in milk fat depression in ruminants [70], the potential effect of CLA isomers on increasing the synthesis of unsaturated fatty acids in milk and the expression of *SCD* in mammary glands that are involved in unsaturated fatty acid synthesis was also observed in the study of Shi et al. [71]. Harvatine et al. [72] also found that the CLA-induced milk fat depression of dairy cows was characterized by an energy-partitioning feather with the increased expression of lipogenic factors *SREBF1*, *PPARγ* and *SCD* in adipose tissue. Therefore, the potential effect of CLA on lipid metabolism might be both tissue- and isomer-specific. Given the significant role of fatty acid profiles in inflammatory processes, further studies should be performed to explore how different CLA isomers connect adipocytokine and inflammation signals.

## 5. Conclusions

**Figure 9:**
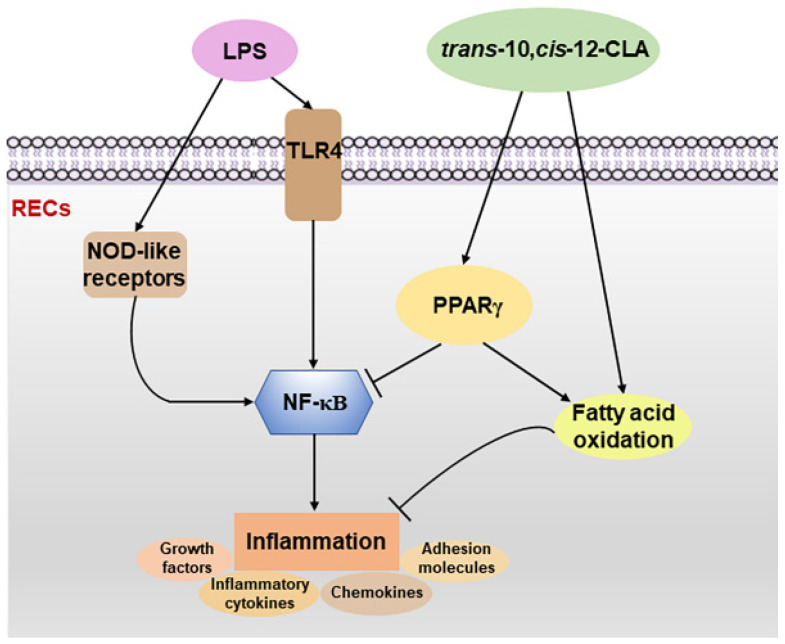
Proposed mechanisms involved in the protective effects of *trans*-10,*cis*-12-conjugated linoleic acid (CLA) on alleviating the inflammatory response in ruminal epithelial cells (RECs) upon lipopolysaccharide (LPS) stimulation. The anti-inflammatory effects of *trans*-10,*cis*-12-CLA on LPS-induced inflammation in RECs may be mediated by the activation of PPARγ, the inhibition of the Nod-like receptor or TLR4-NF-κB-mediated inflammatory responses. Simultaneously, *trans*-10,*cis*-12-CLA enhanced the expression of genes involved in fatty acid oxidation in RECs, which may also be related to the anti-inflammatory effects.

Our in vitro results demonstrated that *trans*-10,*cis*-12-CLA and *cis*-9,*trans*-11-CLA affected inflammatory responses and adipocytokine signaling in RECs in a distinct isomer-specific manner. The 100 μM *trans*-10,*cis*-12-CLA showed better anti-inflammatory effects than *cis*-9,*trans*-11-CLA and may protect RECs against LPS-induced inflammation by activating PPARγ, inhibiting NLRs or TLR4-NF-κB signaling and upregulating the expression of genes involved in the FAO process (Figure 9). As a frequently used feed additive for ruminants, most of the reported benefits of CLA are derived from the mixture of CLA isomers. The present study indicated that each isomer of CLA exerts different biological functions on ruminal epithelium; these results not only provide additional insight to better determine the mechanisms regarding the protective innate immunity of different CLA isomers in the ruminal epithelium and the potential interaction of rumen microbes with innate immune cells in the ruminal epithelium, but also offer important references for determining the practical target use of these dietary fatty acids in animal nutrition to prevent inflammation-related diseases within the rumen. However, in vivo studies are required to confirm these findings.

## Data Availability

The sequences obtained in study were deposited in the NCBI Sequence Read Archive under accession number PRJNA679354.

## Supplementary Materials

The following are available online at https://www.mdpi.com/article/10.3390/ani11041169/s1, Figure S1: Effect of *cis*-9,*trans*-11-CLA pretreatment on the GO terms (**A**) and KEGG pathways (**B**) in RECs upon LPS stimulation, Table S1: Primers used in mRNA abundance analysis.
