# Supplementary material for: Isomer-Specific Effects of cis-9,trans-11- and trans-10,cis-12-CLA on Immune Regulation in Ruminal Epithelial Cells
Source: Animals (Basel). 2021 Apr 19;11(4):1169. doi: 10.3390/ani11041169 (PMC8072642; doi:10.3390/ani11041169)
Supplement: Supplementary file 1 [file animals-11-01169-s001.zip › animals-1118606-supplementary/Supplementary data.pdf]

Isomer-Specific Effects of *cis*-9,*trans*-11- and *trans*-10,*cis*-12-CLA on Immune Regulation in Ruminal Epithelial Cells

Yang et al. Online Supplementary Material

Table S1. Primers used in mRNA abundance analysis.

Gene	NCBI accession	Primer sequence 5′- 3′
TNF-α	NM_001024860	F: GTCCAACTTTAAACAGCTGCACTTA
		R: CCCAAACTTGTGGACCCGA
IL-6	NM_001009392	F: ACACTGACATGCTGGAGAAGATGC
		R: CCGAATAGCTCTCAGGCTGAACTG
NF-κB	XM_027960471	F: ATTGAGCGTCCTGTAACCGTGTTC
		R: TGCACCTCCTCCTTGTCTTCTACC
IL-1β	NM_001009465	F: GCAACCGTACCTGAACCCAT
		R: TGTTGGGTGCAGCTCTTCAT
CX3CL1	XM_027977870	F: AGGCATGAGTGAGCCTGAAGA
		R: TAAGACAGCGCGGGAAAGAG
CCL20	XM_012147649	F: GTCTCCGATACACAGAACGAATACT
		R: TTCACCCACTTCTTCTTTGGATCTG
CD40	XM_012189293	F: AAACAGAATGCCAGTCCTGCGGTAA
		R: ACGCAAGTGGTGTCTGTATTCAAGG
ICAM-1	NM_001009731	F: CAATACATCCTCCAAAAGCCATCAT
		R: CACATCACTCAGTTCAAAAACCTTC
IRF1	NM_001009751	F: ATTGAAGAGGTGAAGGACCAGAGCA
		R: TCGGCTGGACTTGGACTTTCTCTCT
RIPK2	XM_012184164	F: AAAGCGAGGGCTTCTGTCAA
		R: CATCTGGATTTTGCGCCCAC
MMP13	NM_001166179	F: GCCACTTTATGCTTCCTGACGATGA
		R: CGGCGTTTTCGGATGTTTAGAGTTG
CDK17	XM_027967633	F: GAGAAGGCTATCCCTCACGC
		R: CACTCTCGCCATCTGATCCC
WNT10A	XM_004004935	F: AGTCCTATCTTCAGCAGAGGTTTCC
		R: AGGGTGCTCGGGAACTCCGTGGCTC
TLR4	NM_001135930	F: GGTTTCAGGAATGCCACTT
		R: CTTTCACCTCTGCCATACTTT
TICAM1	XM_004023497	F: CACGACACCAGGTCAGCTC
		R: GCCAGTGGACCTTAGGGAAC
PPARγ	NM_001100921	F: GCTGCTCCAGAAAATGACGG
		R: GTGGGTGGAGACTCATGTCC
SCD	NM_001009254	F: TGCTGATCCCCACAATTCCC
		R: TGAAGCACAACAGCAGGACA
FADS2	XM_015103138	F: ACCTCTCTGTTTACAAGAAGTCCAT
		R: TGGAAGATGTTGGGTTTGGCGTGGT

SREBF1	XM_027974781	F: AAACGCTACCGCTCTTCCATCA
		R: TCCACCACCTCGGGCTTCAT
CPT1A	NM_001009414	F: GGCGCCGGGTGCATT
		R: GACTGTGAACTGGAAGGCGA
YWHAZ	NM_001267887	F: GATGAAGCCATTGCTGAACTTGA
		R: CAGCTTCGTCTCCTTGGGTA
GAPDH	NM_001190390	F: GTCTTCACTACCATGGAGAAGG
		R: TCATGGATGACCTTGGCCAG

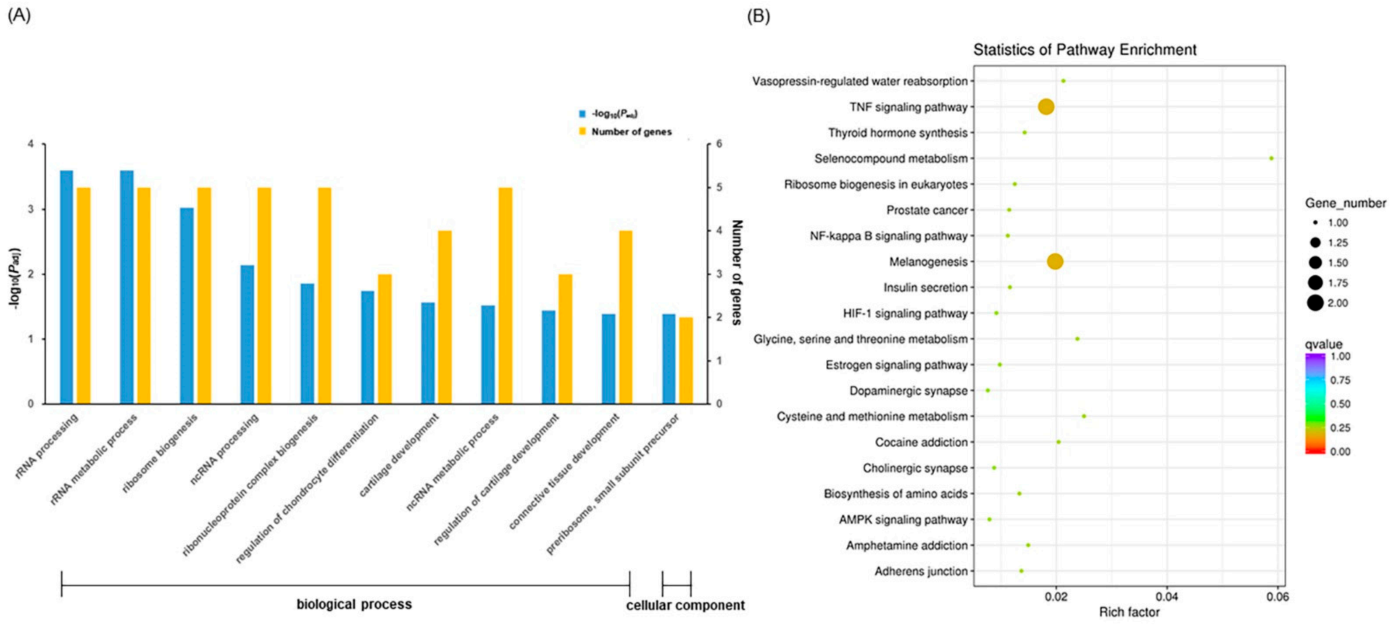

Figure S1. Effect of *cis*-9,*trans*-11-CLA pretreatment on the GO terms **(A)** and KEGG pathways **(B)** in RECs upon LPS stimulation. The DEGs used for functional annotation were significantly downregulated in the *c*-9,*t*-11-CLA+LPS group and upregulated in the LPS group. The circle size indicates the number of enriched genes, and the circle color indicates the P_{adj} value.
